# High Performance Polarization‐Resolved Photodetectors Based on Intrinsically Stretchable Organic Semiconductors

**DOI:** 10.1002/advs.202204727

**Published:** 2022-11-18

**Authors:** Yerun Gao, Jiawen Liao, Haoyu Chen, Haijun Ning, Qinghe Wu, Zhilin Li, Zhenye Wang, Xinliang Zhang, Ming Shao, Yu Yu

**Affiliations:** ^1^ Wuhan National Laboratory for Optoelectronics Huazhong University of Science and Technology Wuhan 430074 P. R. China; ^2^ Department of Chemistry and Key Laboratory for Preparation and Application of Ordered Structural Materials of Guangdong Shantou University Guangdong 515063 P. R. China

**Keywords:** organic photodetectors, polarization‐resolved, stretchable

## Abstract

Polarization‐sensitive photodetectors based on anisotropic semiconductors sense both the intensity and polarization state information without extra optical components. Here, a self‐powered organic photodetector (OPD) composed of intrinsically stretchable polymer donor PNTB6‐Cl and non‐fullerene acceptor Y6 is reported. The PNTB6‐Cl:Y6 photoactive film accommodates a remarkable 100% strain without fracture, exhibiting a high optical anisotropy of 1.8 after strain alignment. The resulting OPD not only shows an impressive faint‐light detection capability (high spectral responsivity of 0.45 A W^−1^ and high specific detectivity of 10^12^ Jones), but also has a high anisotropic responsivity ratio of 1.42 under the illumination of parallel and traversed polarized light. To the best of the authors’ knowledge, both the detector performance and polarization features are among the best‐performing OPDs and polarization‐sensitive photodetectors. As a proof‐of‐concept, polarization‐sensitive OPDs are also utilized to set up a polarimetric imaging system and full‐Stokes polarimeter. This work explores the potential of highly stretchable organic semiconductors for state‐of‐art polarization imaging and spectroscopy applications.

## Graphical abstract

The authors fabricate polarization‐resolved organic photodetectors based on the 100% strained active film, they display impressive faint‐light detection and polarization‐resolved capability. Especially, a high responsivity of 0.45 A W^−1^, specific detectivity of 10^12^ Jones, linear dynamic range of 132 dB, and bandwidth of 21.1 kHz are obtained without bias. Also, a polarization imaging system and full‐Stokes polarimeter is demonstrated.

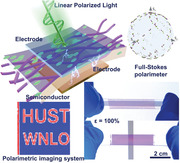

## 1 Introduction

Conventional panchromatic photodetectors measure the intensity of light and provide the spectral information of objects. While the detection of the polarization state or phase of light would give us richer information about surface features, shading and roughness of materials, etc.^[^ ^1^ ^]^ Polarization‐resolved photodetectors, which are capable of detecting both the intensity and phase of light, yield a broad range of applications including polarization division multiplexing communication, remote sensing, quantum optics and medical diagnosis.^[^ ^2^, ^3^, ^4^ ^]^ The standard polarization‐resolved photodetectors require attaching optical polarization components in front of conventional photodetectors or arranging multiple detectors side‐by‐side. Though effective, the extra optical structure substantially increases the complexity, size, and cost of the detection or imaging system. Additionally, it inevitably causes light loss and reduces the responsiveness of the photodetector.

Another alternative strategy to fulfill polarization detection is based on the intrinsic anisotropic optoelectronic properties of semiconductor materials without the need for external optics. A variety of low‐dimensional semiconductors such as one dimensions (1D) nanowires (InP) and two dimensions (2D) materials (black phosphorus, MoS_2_, SbS_2_,GeSe, GeSe_2_) have shown the intrinsic anisotropic light absorption and polarized photoresponse, demonstrating the great potential in polarization‐resolved photodetectors.^[^ ^5^, ^6^, ^7^, ^8^, ^9^, ^10^ ^]^ Unfortunately, the growth of orientated nanowires^[^ ^11^, ^12^ ^]^ and large‐area 2D materials are very challenging. Besides, photodetectors based on low‐dimensional semiconductors usually exhibit large dark current, low detectivity and slow detection speed, which also typically require reverse bias voltage operation. Recently, organic semiconductors have attracted much attention for photodetection applications, owing to their tunable detection wavelength, weak‐light sensitivities, low‐cost and large‐area fabrication. It is known that most semiconducting polymers possess the major optical transition dipole moment (*π*–*π* ^*^) along the conjugated backbone. When the semiconducting polymer is preferentially oriented or aligned, only the polarized light with electric field parallel to the direction of the polymer backbone can be absorbed, resulting in anisotropic optical property. In addition, the reorientation of polymer semiconductors induces anisotropic charge transport. Particularly, the polarization dichroic ratio (DR) relies on the degree of polymer alignment in film. Recently, Conner group successfully aligned poly(benzodithiophene fluorinated benzotriazole) (PBnDT‐FTAZ) donor and poly(naphthalene diimide‐alt‐bithiophene) (N2200) acceptor blend film by combing mechanical rubbing and annealing processes, and subsequently achieved a polarization‐resolved photodetector. However, they found that it is difficult to obtain high responsivity and polarization sensitivity at the same time,^[^ ^13^ ^]^ because the rubbing process scratches the film surface and thus deteriorates the device performance. Uniaxial straining is another alternative method to reorient the film. The degree of alignment of polymer film and in turn their polarization sensitivity linearly increase with the applied strains, but rapidly decrease as soon as passing the fracture strain of film. Thus, the stretchable semiconductors capable of accommodating large tensile strains play a critical role on the polarization characteristics of films. Unfortunately, the crack‐onset strain (COS) of most high‐performance semiconducting polymers is typically less than 5%.^[^ ^14^ ^]^ The tradeoff between optoelectrical and mechanical properties is that rigid conjugated backbones of polymers lead to high charge mobility and excellent optoelectronic performance,^[^ ^15^ ^]^ however, they are mechanically brittle and fragile. Also, the OPDs, similar to organic photovoltaics, consist of polymer electron donors and small molecule or polymer acceptors. Blending highly crystalline small molecules into polymer always leads to a significant decrease of film stretchability even though polymer films are very ductile. Though the polymer acceptor N2200 has been reported to endure a strain as large as 30%,^[^ ^16^ ^]^ the weak absorption coefficient of narrow bandgap N2200 limits the photo‐responsivity for the photodetection applications. Currently, most best performed organic photovoltaics and OPDs are based on the polymer: small molecule blend system, which only endures <20% strain and thus is not able to realize high anisotropic optical polarization via straining alignment.^[^ ^17^ ^]^ Therefore, it is crucial to develop OPDs simultaneously possessing excellent mechanical properties and optoelectronic detection performance.

Herein, we reported a polarization‐resolved OPD by strain aligning an intrinsically stretchable PNTB6‐Cl polymer donor and Y6 small molecular acceptor blend. The PNTB6‐Cl:Y6 blend film shows superior mechanical deformability, which can be stretched up to 100% strain. Encouragingly, the strained OPD exhibit a distinct optical anisotropy of 1.82 and a responsivity anisotropy of 1.42 under the illumination of parallel and traversed polarized light. Moreover, the strain‐aligned OPDs also show excellent device performance in terms of high responsivities of 0.33 A W^−1^ at 585 nm and 0.45 A W^−1^ at 815 nm, as well as a high specific detectivity of over 10^12^ Jones. To the best of our knowledge, this is the highest detectivity reported for intrinsically polarization‐resolved photodetectors.^[^ ^13^, ^16^, ^18^, ^19^, ^20^, ^21^, ^22^, ^23^, ^24^, ^25^, ^26^, ^27^, ^28^, ^29^, ^30^, ^31^, ^32^, ^33^ ^]^ Further, we demonstrated a self‐powered polarization imaging system and a full‐Stokes polarimeter. Our results reveal that stretchable organic semiconductors are very promising for the future development of high‐performance polarization‐sensitive photodetectors.

## 2 Results

### 2.1 Optical and Mechanical Properties of Stretchable Organic Semiconductors

**Figure 1:**
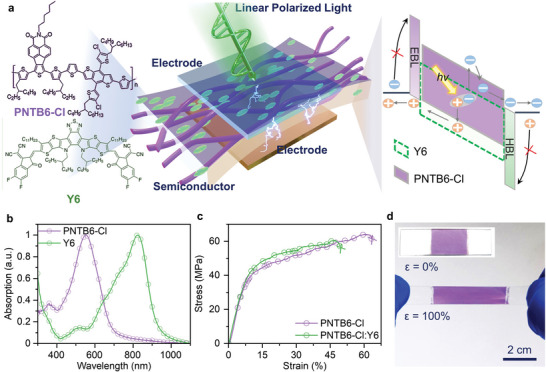
a) Schematic of polarization‐resolved photodetector. The left part is the chemical structures of PNTB6‐Cl and Y6, the right part is energy band diagram of the photodetector under light illumination. b) Normalized absorption spectra of PNTB6‐Cl film and Y6 film. c) Stress–strain curves of PNTB6‐Cl and PNTB6‐Cl:Y6 LBL film using pseudo‐free standing tensile test. d) Pictures of the pristine film and 100% strained film on PDMS substrates.

We fabricated the polarization‐resolved photodetectors based on the highly strained organic semiconductors. As shown in **Figure** **1**a, the photo‐sensing layer sandwiched between the two electrodes of OPD comprises of polymer electron donor (abbreviated PNTB6‐Cl) and small molecular acceptor Y6. Especially, PNTB6‐Cl donor polymer, which is a naphthalenothiophene imide (NTI) based polymer grafted with linear alkyl chains, was home‐synthesized based on our previous publications.^[^ ^34^ ^]^ The right part shows the energy band diagram of OPD. In the dark, the electron blocking layer (EBL) MoO_3_ and hole blocking layer (HBL) ZnO substantially raise the injection barrier. Under illumination, the photo‐sensing layer absorbs photon and then generates excitons. Then, the photogenerated excitons are dissociated into free carrier at the PNTB6‐Cl/Y6 interface, and drifted to the corresponding electrode under reverse bias. The absorption spectra of active materials determine the detection wavelength range of OPD. As seen from Figure 1b, the novel PNTB6‐Cl exhibit a strong absorption band in the visible light region (400–670 nm) with a peak at 552 nm, while non‐fullerene acceptor Y6 possesses strong absorption in the near‐infrared region (500–900 nm) with an absorption peak at 825 nm. Thanks to their complementary absorption, the absorption of the OPD sensing layer (PNTB6‐Cl:Y6) covers a wide spectral range from 400 to 900 nm. Note PNTB6‐Cl and Y6 have different solubility in chloroform, we are able to form a pseudo‐bilayer structure using layer‐by‐layer (LBL) process.^[^ ^34^ ^]^ Compared to conventional bulk heterojunction structure, LBL process has been proved as an effective method to control the vertical phase separation structure and thus influence the device performance.^[^ ^35^ ^]^ In order to obtain the polarization photosensing capability, the active layer should have good mechanical stretchability for strain alignment. Next, the mechanical parameters of active layer are characterized by two different methods: film‐on‐elastomer and film‐on‐water. First, the PNTB6‐Cl film with a thickness of 52 nm was transferred onto the PDMS elastomer and then continuously increased strain until the formation of cracks. Surprisingly, the PNTB6‐Cl film shows a remarkable COS of 70% observed from the optical microscopy (Figure S1, Supporting Information). Second, to better reflect the intrinsic mechanical properties of materials, the freestanding PNTB6‐Cl film possessed with the standard “dog‐bone” structure is floated on the water, known as “pseudo free‐standing tensile test”, avoiding the possible substrate impact. Figure 1c confirms that the PNTB6‐Cl film can be stretched up to 63% before it fractures. In comparison, most reported conjugated polymers typically have limited stretchability (*ε* < 5%).^[^ ^14^ ^]^ The slight difference in COS measured between two methods can be ascribed to the influence of the substrate. In addition, the Young's modulus of PNTB6‐Cl film is 0.46 GPa extracted from the linear region of stress–strain curves, indicating its soft nature. The measured Young's modulus (*E*_f_) is almost half value of widely used PM6 and PBDB‐T (*E*_f_ ≈ 0.84 GPa) polymers. We hypothesize that the superior tensile properties of PNTB6‐Cl may originate from its unique twisted backbone structure, which is distinct from the planar structure of normal PM6 donor materials. The twisted backbone structure of PNTB6‐Cl leads to stronger polymer chain entanglement and thus allows the polymer chain to absorb more stress.^[^ ^36^ ^]^ Moreover, the twisted molecular structure substantially reduces the film crystallinity, which is beneficial for improving the ductility of polymers.^[^ ^37^ ^]^ Therefore, the film can be strained to 100% on a PDMS substrate with no obvious cracks (Figure 1d).

Furthermore, it is very common to observe the rapid decrease of COS when incorporating small molecules PCBM or non‐fullerene acceptors into polymer donors, even though the polymer itself has good ductility. For example, P3HT shows a COS of over 6% while the P3HT:PCBM blend shows a low COS of 2%, and PM6 shows a COS of 12% but a PM6:Y6 blend film has a lower COS of 5%.^[^ ^18^, ^36^, ^38^ ^]^ Encouragingly, the PNTB6‐Cl:Y6 blend film prepared by the LBL processing shows an impressive COS of ≈50%. As illustrated in Figure S2, Supporting Information, the blend film can endure an enormous strain of 100% without fracture. The Young's modulus of blend film only slightly increases to 0.5 GPa, suggesting that the incorporation of Y6 small molecule acceptor does not cause the film stiffening. The vertical phase structure of PNTB6‐Cl:Y6 film is critical to the performance of OPDs. Figure S3, Supporting Information, shows the film‐depth‐dependent absorption spectra of PNTB6‐Cl:Y6, from which the volume ratio of PNTB6‐Cl and Y6 along the film thickness can be calculated. This reveals a pseudo‐bilayer structure that the upper surface (5 nm) of blend film is dominated by Y6, and the underneath film is enriched with PNTB6‐Cl. The reduced ratio of highly crystalline Y6 is in favor of the mechanical stretchability of the blend film. Therefore, the superior stretchability of PNTB6‐Cl and Y6 blend film allows strain aligning to realize the polarized light detection.

### 2.2 Optoelectrical Performance of Organic Photodetectors

**Figure 2:**
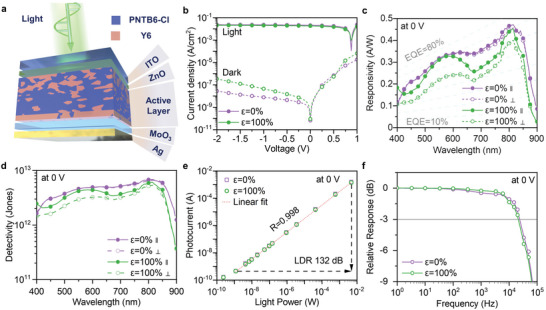
a) Schematic diagram of the photodetector structure. b) Current–voltage curves of detectors with the film pristine and 100% strained in dark and under white illumination (AM1.5G solar spectrum). c) The responsivity under zero bias of the detectors under linear polarized light parallel (∥) and perpendicular (⊥) to the strain direction with the film pristine and 100% strained. d) Specific detectivity under zero bias as a function of light wavelength at the EQE measurement frequency (≈225 Hz) of detectors with pristine film and 100% strained film. e) Measured irradiance‐dependent photocurrent under zero bias of 0% and 100% strained devices. f) Frequency response of the photodetectors operated under zero bias.

Next, we fabricated the intrinsically stretchable OPD based on the PNTB6‐Cl:Y6 active layer and characterized device performance parameters. **Figure** **2**a illustrates the device architecture: indium tin oxide (ITO)/zinc oxide (ZnO)/PNTB6‐Cl:Y6/molybdenum oxide (MoO_3_)/silver (Ag). ZnO is chosen as the electron transport and HBL, due to a suitable conduction band energy level of −4.07 eV for electron transport and a valence band energy level of −7.37 eV for efficient hole blocking, while MoO_3_ was used as electron transport and HBL. The dark current density (*J*_dark_) of the device can be significantly reduced after choosing appropriate charge selection layers and increasing active film thickness. Next, the strained PNTB6‐Cl:Y6 active layer with a thickness of 74 nm is transferred onto the ITO/ZnO substrate. The detailed fabrication process is shown in Figure S4, Supporting Information. There is an energy barrier ≈0.61 eV (Figure S5, Supporting Information) between the lowest unoccupied molecular orbital (LUMO) of the PNTB6‐Cl donor and Y6 acceptor, which could prevent the thermally generated electrons transferring from acceptor to donor at low bias. Thus, the *J*_dark_ is expected to be effectively suppressed from this pseudo‐bilayer configuration formed by the LBL processing.

Figure 2b shows the current density–voltage curves of OPDs with unstrained (*ε* = 0%) and strained (*ε* = 100%) active layer. The *J*_dark_ of unstrained OPD is 2.82 × 10^−8^ A cm^−2^ under a reverse bias of −2 V. Considering a low *J*_dark_ is normally achieved from thick films (thickness > 300 nm), this low *J*_dark_ obtained from our thin active film (≈74 nm) is very impressive, indicating a high quality of active layer and charge selection layers. Although *J*_dark_ can be further suppressed by simply increasing the thickness of the active layer (Figure S6, Supporting Information), a thick active layer would absorb the light from different polarization states equivalently, resulting in a low polarization anisotropy. Here, we tried to maximize the anisotropy of polarization‐resolved photocurrent in the meantime retaining a low *J*_dark_ and thus high detectivity. Notably, the *J*_dark_ of a 100% strained device remains at a low value of 4.52 × 10^−7^ A cm^−2^. This comparable *J*_dark_ implies that the optoelectronic properties of the active film do not degrade after the strain aligning. It should be mentioned the OPDs show a low *J*_dark_ (<100 pA cm^−2^) at 0 V, which is three orders of magnitude lower than the value under −2 V bias voltage. Also, the photocurrent density (*J*_sc_ ≈ 18 mA cm^−2^) at 0 V is comparable to the current density (*J*_sc_ ≈ 21 mA cm^−2^) under −2 V, indicating sufficient charge extraction at 0 V. Therefore, the ultra‐low dark current and high photocurrent at 0 V indicate our OPD can be operated in self‐powered mode.

Figure 2c represents the responsivity (*R*) spectra of OPDs illuminated under linear polarized light parallel (*R*_∥_) and perpendicular (*R*_⊥_) to the direction of applied strain. The peak responsivity of unstrained device is 0.33 A W^−1^ at 575 nm and 0.47 A W^−1^ at 815 nm. In addition, the external quantum efficiency (EQE) of OPDs under zero bias is over 70% in the range of 400–900 nm, consistent with its high responsivity. To the best of my knowledge, this large *R*_∥_ is among the state‐of‐the‐art organic photodetectors.^[^ ^39^ ^]^ For an unstrained device, the anisotropic response ratio (*R*_∥_/*R*_⊥_) is close to 1, indicating an isotropic film. In contrast, for the 100% strained device, the *R*_∥_ is 0.33 A W^−1^ at 575 nm and 0.45 A W^−1^ at 815 nm, while the *R*_⊥_ is 0.23 A W^−1^ at 575 nm and 0.4 A W^−1^ at 815 nm, corresponding to a large *R*_∥_/*R*_⊥_ of 1.42 at 575 nm. This result indicates the anisotropic responsivity is related to the PNTB6‐Cl donor instead of a Y6 acceptor. More importantly, the high *R* and *R*_∥_/*R*_⊥_ ratio are achieved simultaneously, benefiting from the superior optoelectronic and mechanical ductility of the active layer. Furthermore, the specific sensitivity (*D* ^*^), which reflects the sensitivity of photodetectors, is determined by the following equation:

(1) $$D^{*}=\frac{R\sqrt{A}}{S_{n}}$$

where *A* is the light‐sensitive area (0.045 cm^2^), *S*_n_ is the noise current spectral density in amperes per half hertz. As shown in Figure S7, Supporting Information, the *S_n_* is 1.46 × 10^−14^ A Hz^−1/2^ for unstrained devices and 1.58 × 10^−14^ A Hz^−1/2^ for 100% strained devices measured at EQE frequency (≈225 Hz). Figure 2d shows the calculated *D* ^*^ as a function of the wavelength. The *D* ^*^ of the unstrained OPD is over 10^12^ Jones across the spectral detection range (400–900 nm) and reaches the maximum of 6.85 × 10^12^ Jones at 815 nm. Encouragingly, the *D* ^*^ of a 100% strained device does not decrease upon strain aligning. It shows a high *D* ^*^ of 6.1 × 10^12^ Jones and 5.4 × 10^12^ Jones at 815 nm under parallel and perpendicular polarized light illumination, respectively. The slightly decreased *D* ^*^ under 100% large strain is likely due to the decreased mobility as shown in Figure S8, Supporting Information. To note, this *D* ^*^ is comparable to the state‐of‐art commercial Si photodetector (Hamamatsu S1133, D^*^ ≈ 2 × 10^12^ Jones).^[^ ^39^ ^]^ Furthermore, we measure the linear dynamic range (LDR) and −3 dB frequency (*f*_−3 dB_) of our OPD. Figure 2e shows the photocurrent density of 0% and 100% strained devices as a function of the incident light power density under a high power LED irradiation (*λ* = 525 nm). The strained devices show a similar LDR and *f*_−3 dB_ to the unstrained devices. The minimum power density in the linear range is 1.18 × 10^−9^ W and the maximum value is 4.68 × 10^−3^ W, corresponding to a LDR of 132 dB at 0 V. As seen from the frequency‐dependent responsivity (Figure 2f), the *f*_−3 dB_ measured under 0 V is 23.7 kHz and 21.1 kHz for 0% and 100% strained devices, respectively. The high response speed of OPDs is sufficient for polarization imaging applications. Recently, the emerging 2D materials such as black phosphorus (b‐P) and transition metal dichalcogenides (MoS_2_, ReS_2_, ReSe_2_, GeSe_2_, etc.) have been demonstrated for the polarization‐sensitive photodetectors.^[^ ^5^, ^6^, ^7^, ^8^, ^40^, ^41^, ^42^ ^]^ However, most 2D materials‐based polarization photodetectors show comparatively low LDR (<60 dB) and low respond speed (<1 kHz). Additionally, those detectors are typically required to operate under reverse bias. By contrast, our OPDs operating at 0 V bias outperform reported 2D material based photodetectors in terms of detectivity, response speed, and power consumption.

### 2.3 Polarization‐Resolved Organic Photodetectors

**Figure 3:**
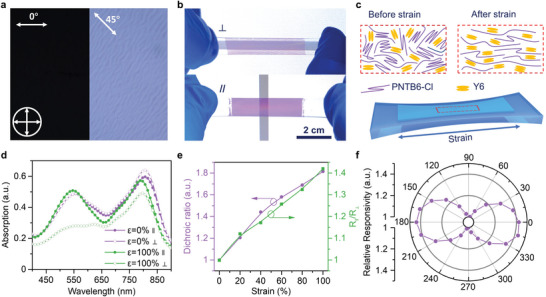
a) Cross‐polarized optical microscopy images of 100% strained PNTB6‐Cl:Y6 film. The crossed arrows stand for the orientation of the cross‐polarizers, and the white arrows indicate the stretching direction. b) Pictures of the 100% strained film with film orientation horizontal (bottom) and vertical (top) in front of a liquid crystal display. c) Schematic of polymer chain and small molecular in pristine film and strained film. d) Absorbance spectra of PNTB6‐Cl:Y6 LBL film under linear polarized light parallel (∥) and perpendicular (⊥) to the strain direction of pristine (0%) and 100% strained film. e) The dichroic ratio and the responsivity anisotropy at 535 nm with strain varies from 0% to 100%. f) Relative responsivity of 100% strained device as a function of linear polarization angle (illuminated by a 532 nm laser).

Furthermore, the strain‐oriented OPDs also demonstrated polarization‐sensitive photodetection. **Figure** **3**a displays the cross‐polarized optical microscopy (CPOM) images of 100% strained PNTB6‐Cl:Y6 film. When the incident polarized light is perpendicular to the strain direction (0°), the light extinction was observed from the cross‐polarized optical microscopy (CPOM). As the sample film rotates from 0° to 45°, the images gradually become brighter, indicating good arrangement of the whole film. Also, when we place the strained film horizontally and vertically to the polarizer of the LCD screen, the strained active film shows the prominent anisotropic light transmission (Figure 3b). These phenomena unambiguously imply the high degree of the oriented molecules in our strained active film. Figure 3c depicts the schematic of the polymer and small molecular blend film without (w/o) and with stretching. Without straining, the polymer donors possess random chain orientation and exhibit isotropic light absorption. When the strain is applied, the polymer and small molecular are aligned along the strain direction, resulting in optical anisotropy. We further quantify the degree of molecules alignment under strain by measuring the absorbance anisotropy. Figure 3d represents the absorption spectra of 0% and 100% strained PNTB6‐Cl:Y6 film under linear polarized light parallel and perpendicular to the strain direction. In particular, the dichroic ratio (DR) is defined as the film absorbance ratio of light with polarization parallel (∥) and perpendicular (⊥) to the orientation direction. Figure S9, Supporting Information, displays the DR spectra of 0% to 100% strained films. The DR at 535 nm corresponding to PNTB6‐Cl gradually increases from 1 to 1.84 upon the applied strain, indicating the progressive chain alignment of the PNTB6‐Cl polymer donor. The DR at 774 nm related to Y6 acceptor reaches a maximum value of 1.2 under 100% strain, suggesting that Y6 is reorientated during strain process. The anisotropic optical absorption eventually results in photocurrent and responsivity anisotropy. Figure S10, Supporting Information, represents the *R*_∥_/*R*_⊥_ of strain‐oriented devices responsivity by varying strains from 0 to 100% (detailed in Figure S11, Supporting Information). Figure 3e exhibits that the *R*_∥_/*R*_⊥_ increases with the applied strains, consistent with the DR change. The *R*_∥_/*R*_⊥_ of the 100% strained device reaches a high value of 1.42 at 535 nm. To note, this high anisotropic *R* was achieved without any other post‐treatment such as mechanical rubbering and high‐temperature annealing process.^[^ ^16^ ^]^ Furthermore, the *R*_∥_/*R*_⊥_ can be further amplified to 22 by combining a field‐effect transistor (FET) and a resistor. In the circuit structure (Figure S12, Supporting Information), the photocurrent under the illumination of polarized light generates different voltages across the resistor. Accordingly, the gate electrode voltage of FET changes and enables the FET to operate in the subthreshold region.^[^ ^43^ ^]^ As a result, the current change across the source and drain electrodes will be amplified by several times, compared to the photocurrent directly measured from OPDs.

Figure 3f also illustrates the normalized polar curves of the responsivity as a function of the polarization angle under linear polarized light illumination. Since the intensity variation of incident light is <1% (Figure S13, Supporting Information), we are able to exclude the influence of the incident optical power on the photocurrent response. The maximum responsivity appears along the strain direction, implying that the polarization axis of the detector can be precisely controlled by the strain direction. Moreover, Table S1, Supporting Information, summarizes the detectivity and *R*_∥_/*R*_⊥_ of polarization‐resolved low‐dimensional and organic photodetectors reported to date.^[^ ^13^, ^16^, ^18^, ^19^, ^20^, ^21^, ^22^, ^23^, ^24^, ^25^, ^26^, ^27^, ^28^, ^29^, ^30^, ^31^, ^32^, ^33^ ^]^ To the best of our knowledge, our devices exhibit the best detectivity (>10^12^ Jones), and it is worth mentioning other merits like LDR and *f*_−3 dB_ are among the best‐performed polarization‐resolved photodetectors.

### 2.4 Applications: Polarimetric Imaging System and Full‐Stokes Polarimeter

**Figure 4:**
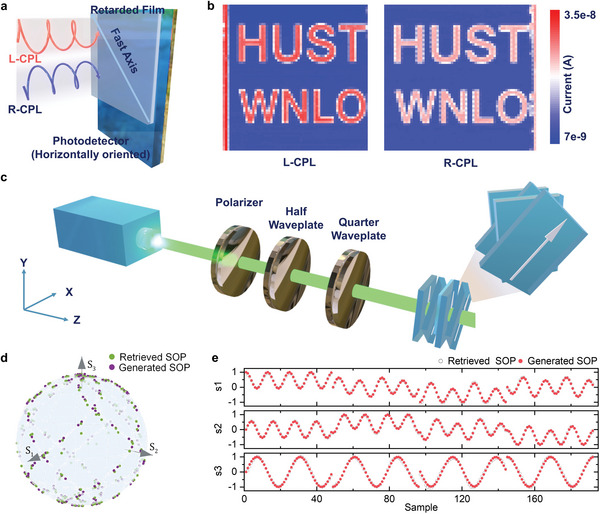
a) Schematic illustration of the structure of circular polarized light detector. b) Pictures of the letters “HUST WNLO” imaged from left‐handed circularly polarized light and right‐handed circularly polarized light. c) Experimental setup of the Stokes parameters test. d) The retrieved SOPs and generated SOPs on the Poincaré Sphere. e) The retrieved Stokes parameters and generated Stokes parameters.

Apart from the linear polarized light detection, we also demonstrate circularly polarized light detection by combining our linearly polarized light detector with a *λ*/4 polymeric retardation film. **Figure** **4**a displays the schematic of a circularly polarized light detector, in which the fast axis of the retardation film is aligned at a 45° angle to the film strain direction. Thus, the left‐handed circular polarized light (L‐CPL) is converted to the linear polarized light parallel to the strain direction, and the right‐handed circularly polarized light (R‐CPL) is converted into linearly polarized light perpendicular to the strain direction. Figure S14, Supporting Information, displays the distinct responsivity spectra (400–900 nm) of the circularly polarized light detector under L‐CPL and R‐CPL illumination. To evaluate the capability of the detector to resolve circularly polarized light, the anisotropy factor (*g*_res_) is calculated with the formula:

(2) $$g_{\mathrm{res}}=\frac{2\left(R_{L}-R_{R}\right)}{\left(R_{L}+R_{R}\right)}$$

where *R*_L_ and *R*_R_ stand for the responsivity of devices under L‐CPL and R‐CPL illumination, respectively. The *g*_res_ exhibits a maximum value of 0.38 at 545 nm, indicating sufficient contrast for circular polarization imaging.

First, we proceed to demonstrate a single‐pixel polarimetric imaging system, which simultaneously detect both the intensity and polarization information of light. The layout and experimental setup of the polarimetric imaging system is shown in Figure S15, Supporting Information. The modulated photocurrent signals are acquired by progressively moving the mask patterned with the “HUST WLNO”. To note, our polarized detector has provided a stable detection signal during the date acquisition, implying excellent performance stability of polarized photodetector. Figure 4b presents the images composed of 64 × 64 array pixels under the L‐CPL and R‐CPL illumination, respectively. Note that the picture imaged under L‐CPL shows higher contrast than the picture imaged under R‐CPL, implying the great potential for the circularly polarimetric imaging system.

Second, we build a full‐Stokes polarimeter based on the demonstrated polarization‐resolved OPDs by tightly stacking three linear polarization photodetectors and one circular polarization photodetector (Figure 4c). This novel polarimeter features a compact design, a high measuring speed and improved stability. Generally, the state of polarization (SOP) is described by stokes vector *S* = (*S*_0_,*S*_1_,*S*_2_,*S*_3_)^*T*^. By measuring the current *I*_1_,*I*_2_,*I*_3_,*I*_4_ of the four photodetectors simultaneously, the stokes vector *S* is recovered with *S* = *W*† × *I*, *where* *I* = (*I*_1_,*I*_2_,*I*_3_,*I*_4_)^*T*^, *W* is a 4 × 4 measurement matrix, † means the pseudoinverse of a matrix. Each row of W is a 1 × 4 vector determined by the configuration of each polarization detector.^[^ ^44^ ^]^ In the proposed polarimeter, the film orientation angles of four photodetectors with respect to the *y*‐axis are 0°, 45°, 0° and 60°, corresponding to four vectors: *V*_1_ = *R*_1_ × (1, *ε*, 0, 0)^*T*^, $V_{2}=R_{2}\times {\left(1,\varepsilon /\sqrt{2},\varepsilon /\sqrt{2},0\right)}^{T}$, *V*_3_ = *R*_3_ × (1, 0, 0, *ε*)^*T*^ and *V*_4_ = *R*_4_ × (1, 0, 0.866*ε, −*ε*/2)^*T*^, where *R*_1_, *R*_2_, *R*_3_ and *R*_4_ is the *R*_∥_ of four photodetectors, *ε* is a coefficient between 0 and 1, relating to the DR of OPDs:

(3) $$\varepsilon =1-\frac{1}{DR}$$

Thus, the measurement matrix W of the proposed polarimeter is:

(4) $$W=\left(\begin{array}{cccc} R_{1} & 0 & 0 & 0\\ 0 & R_{2} & 0 & 0\\ 0 & 0 & R_{3} & 0\\ 0 & 0 & 0 & R_{4} \end{array}\right)\left(\begin{array}{cccc} 1 & \varepsilon & 0 & 0\\ 1 & \frac{\varepsilon }{\sqrt{2}} & \frac{\varepsilon }{\sqrt{2}} & 0\\ 1 & 0 & 0 & \varepsilon \\ 1 & 0 & 0.866\times \varepsilon & -\varepsilon /2 \end{array}\right)$$

Before testing, a group of known SOPs is used to calibrate the measurement matrix W:

(5) $$W=\left[I_{1},I_{2},I_{3},\cdot \cdot \cdot I_{n}\right]*{\left[S_{1},S_{2},S_{3},\cdot \cdot \cdot S_{n}\right]}^{†}$$

where *S_n_* is the Stokes vector of the n^th^ known SOP and *I_n_* is the measured photocurrent under the nth known SOP. To characterize the accuracy of the demonstrated polarimeter, 192 SOPs spreading over the Poincaré sphere are generated and measured. As shown in Figure 4d, the retrieved SOPs show a good agreement with the generated SOPs. The measured and generated s1, s2 and s3 are shown in Figure 4e, and the root‐mean‐square (RMS) deviations are 0.040, 0.039 and 0.072, respectively. The RMS deviation of degree of polarization is 5.43%, and the RMS deviations of the azimuth angle and ellipticity angle are 0.899° and 0.914°, respectively (details in Figure S16, Supporting Information).

Our polarimeter shows a good accuracy compared with the literatures (0.050, 0.048 and 0.067 as the minimum deviations of s1, s2 and s3).^[^ ^45^, ^46^, ^47^, ^48^, ^49^ ^]^ Also, the polarimeter shows a high sensitivity and a wide dynamic range. Moreover, it is a self‐powered system since no voltage is applied to the photodetectors. The high speed, large dynamic range, lower power consumption, great accuracy and stability validate its practical utility and pave the way for the implementation in a wide range of polarization‐related applications, such as polarization imaging, spectroscopic ellipsometry, astronomy and so on.

## 3 Conclusion

In conclusion, we report a novel polarization‐resolved OPD based on PNTB6‐Cl:Y6 blend active film. Especially, the OPD presents a broad detection range from 400 to 900 nm. Moreover, the OPDs exhibit a high specific detectivity of 6.1 × 10^12^ Jones and responsivity of 0.45 A W^−1^, as well as LDR of 132 dB and −3 dB bandwidth of 21.1 kHz. The high detectivity can be related to the substantially reduced noise current (<100 pA cm^−2^ at 0 V) of PNTB6‐Cl:Y6 blend film processed with LBL method. Furthermore, the PNTB6‐Cl:Y6 blend film exhibit a remarkable intrinsic stretchability up to 100% strain, resulting in distinct optical and photocurrent anisotropy. With the strain‐oriented active film, the OPD displays a unique polarization light detection capability, exhibiting a high *R*_∥_/*R*_⊥_ of 1.42. Benefitting from our polarization‐resolved OPDs, we successfully demonstrate high performance polarimetric imaging system and full‐Stokes polarimeter. Our study points toward the new possibilities of intrinsically stretchable organic semiconductors in developing high‐performance polarization‐sensitive photodetectors.

## 4 Experimental Section

### Materials

The PNTB6‐Cl was synthesized using the process reported previously.^[^ ^34^ ^]^ Y6 was purchased from Solarmer Material. Octadecyltrimethoxysilane (OTMS, 90%) and poly(sulfonate styrene) sodium salt (PSS‐Na) (*M*_w_ ≈ 70 000) was purchased from Aladdin. Poly(dimethyl siloxane) (PDMS, Sylgard 184) was purchased from Dow Corning. WP140HE retardation film was purchased from Edmund Optics.

### Solution Preparation

PNTB6‐Cl solution was prepared with a concentration of 8 mg mL^−1^ in chlorobenzene and stirred at 110 °C for 2 h. Y6 solution was prepared with a concentration of 8 mg mL^−1^ in chloroform. PSS‐Na was dissolved in deionized water (DI water) at the ratio of 0.5 wt%. Sol‐gel zinc oxide precursor solution was prepared according to the previous reported methods.^[^ ^16^ ^]^ OTMS solution was prepared by dissolving OTMS in trichloroethylene at a volume ratio of 0.1%.

### Device Fabrication

ITO substrates and glasses were cleaned by ultrasonication in DI water, ITO cleaning agent, acetone, and ethanol for 30 min each. Plasma was used to clean the substrates for 60 s, solution of OTMS was spin‐coated on a glass substrate at 3000 rpm for 10 s. The OTMS films were treated with NH_3_ in a closed glass jar for 10 min. Then all the substrates were transferred to a glovebox filled with nitrogen and baked at 110 °C for 2 min, then the PNTB6‐Cl and Y6 solution was spin‐coated layer‐by‐layer (LBL) at 3000 rpm for 40 s. The strip‐shaped PDMS stamp was used for transferring the film, PDMS stamp was prepared at a 10:1 weight ratio to crosslinker and cured in a vacuum oven at 60 °C. The transferred films were stretched with PDMS and transferred to PSS‐covered substrates under different strains. PSS solution was spin‐coated on glass at 3000 rpm for 40 s and baked at 150 °C for 10 min to form PSS‐covered substrates. The transferred film on PSS‐Na was immersed in DI water where the PSS‐Na was dissolved, leaving the LBL film freestanding on water.^[^ ^50^ ^]^ A zinc oxide precursor solution was spin‐coated on ITO substrate at 4000 rpm for 60 s and annealed at 200 for 1 h. After the ZnO substrates cooled to room temperature, it was used to pick up the film in water. After natural air drying, the film was transferred to the vapor deposition chamber, for normal device, 10 nm MoO_3_ and 60 nm Ag was evaporated through the mask to form electrodes, for the semitransparent device, 10 nm Au was evaporated instead of 60 nm Ag.

### Pseudo Free‐Standing Tensile Test

First, the PNTB6‐Cl solution was spin‐coated on a PSS‐Na covered substrate, and a nano‐second laser was used to cut the film into a dumbbell shape. Then the dumbbell shape film was floated by dissolving the PSS‐Na with DI water. An Al grip with PDMS stamp was used to grip the sample through van der Waals adhesion. The tensile test was performed by a motorized uniaxial stage with the speed of 50 µm s^−1^ and a micro strain gauge was used to obtain stress value.

### Film and Device Characterization

All microscope photos were taken by a Sunny RX50M microscope. Film‐depth‐dependent absorbance spectra were measured with a Flame‐T fiber spectrometer combining with the plasma etching process. Anisotropic absorbance spectra were obtained using a tungsten lamp, a polarizer, and a Flame‐T fiber spectrometer. The current–voltage curve was measured by a Keithley 2400 source meter under a 100 mW cm^−2^ simulated AM1.5G spectrum white light from an Enlitech SS‐X solar simulator. The dark current–voltage curve was measured by Keithley 2634B dual source meter with a probe station. The EQE and responsivity was measured using a chopped Zolix xenon arc lamp light source, Omni‐*λ*300i monochromator, SR570 current amplifier, and SR830 DSP lock‐in amplifier. The polarization was controlled by a polarizer and a half‐wave plate. Noise current density spectra was measured with SR830 DSP lock‐in amplifier, for which the gain and the sensitivity was set to be 10^8^ V A^−1^ and 100 nV fA^−1^, respectively. To suppress the powerline noise, the notch filter was switched on. The noise current measured under each frequency was collected after 10 s of stabilization for an accurate estimation of the noise. LDR was measured with a 525 nm LED focused with lens, light power was calibrated by HAMAMATSU S2387‐66R photodetector. −3 dB bandwidth test was taken using a high power 525 nm LED modulated by a function generator.

### Single Pixel Imaging System

The imaging system mainly consists of one photodetector and one two‐axis motorized stage, the pixel size was determined by the minimum step size of the motorized stage (≈36 µm), and the array area was determined by the maximum displacement of the motorized stage (100 × 50 cm^2^). The 532 nm laser, linear polarizer, and quarter waveplate were used to generate L‐CPL and R‐CPL. The mask with letters “WNLO HUST” was the object to be imaged. The two‐axis motorized stage was used to hold the mask and move with a step of 0.3 mm. The polarization detector was placed behind the mask and a Keithley 2400 source meter was used to measure the device current. By combining the current signal obtained in each displacement, a 64 × 64 pixels picture was acquired.

### Full‐Stokes Polarimeter

The 532 nm laser was first polarized by a polarizer. Then, the rotating *λ*/2 waveplate and *λ*/4 waveplate were used to generate a group of known SOPs that spread across the Poincaré sphere's surface. The polarimeter was placed behind the *λ*/4 waveplate to measure the generated SOP. The Keithley 2400 was used to measure the photocurrent of the four detectors of the polarimeter. By multiplying the matrix W† and the photocurrent, the SOPs were retrieved.

## Conflict of Interest

The authors declare no conflict of interest.

## Author Contributions

Y.G. and J.L. authors contributed equally. Y.G. and J.L. designed and performed the experiments, carried out data analysis and contributed equally to this work. H.C., Z.L., and Z.W. assisted in device testing. H.N. and Q.W. synthesized the materials. M.S. and Y.Y. directed this project. All authors discussed the results and contributed to the manuscript.

## Supporting information

Supporting Information

## Data Availability

The data that support the findings of this study are available on request from the corresponding author. The data are not publicly available due to privacy or ethical restrictions.
