# Supplementary material for: High Performance Polarization‐Resolved Photodetectors Based on Intrinsically Stretchable Organic Semiconductors
Source: Adv Sci (Weinh). 2022 Nov 18;10(2):2204727. doi: 10.1002/advs.202204727 (PMC9839839; doi:10.1002/advs.202204727)
Supplement: Supplementary file 1 — Supporting Information [file ADVS-10-2204727-s001.pdf]

Supporting Information

High performance polarization sensitive photodetectors based on intrinsically stretchable organic semiconductors

Yerun Gao,[#1] Jiawen Liao,[#1] Haoyu Chen,[1] Qinhe Wu,[*2] Zhilin Li,[1] Zhenye Wang,[1] Xinliang Zhang,[1] Ming Shao[*1] and Y. Yu[*1]

[1]Wuhan National Laboratory for Optoelectronics, Huazhong University of Science and Technology, Wuhan 430074, P. R. China

[2]Department of Chemistry and Key Laboratory for Preparation and Application of Ordered Structural Materials of Guangdong, Shantou University, Guangdong 515063, China.

Corresponding Authors[*]:
Prof. Ming Shao and Prof. Yu Yu
Wuhan National Laboratory for Optoelectronics,
Huazhong University of Science and Technology, Wuhan 430074, China
Prof. Qinhe Wu
Department of Chemistry and Key Laboratory for Preparation and Application of Ordered Structural Materials of Guangdong,
Shantou University, Shantou 515063, China.
Correspondence and requests for materials should be addressed to M. S, Q. W or Y. Z
Email: mingshao@hust.edu.cn; or wuqh@stu.edu.cn; yuyu@mail.hust.edu.cn
#These authors contributed equally: Yerun Gao, Jiawen Liao

Table of Content

Figure S1. Microscope image of PNTB6-Cl film stretched under 70% strain................1

Figure S2. Microscope image of PNTB6-Cl film stretched under 100% strain..............2

Figure S3. Film-depth-dependent light absorption..3

Figure S4. Manufacturing process of polarization organic photodetector……………...4

Figure S5. Schematic diagram of the energy levels of the materials in photodetector.....5

Figure S6. The dark current density of device with different thickness……………..…6

Figure S7. Measured total noise spectra density……………………………………...7

Figure S8. Current-voltage curves of hole-only devices………………………………8

Figure S9. Dichroic ratio spectra of PNTB6-Cl:Y6 LBL film………………………...9

Figure S10. The responsivity anisotropy spectra of the film……………………….....10

Figure S11. The responsivities of the detectors under linear polarized light……….....11

Figure S12. Structure schematic diagram of the polarization-sensitive amplification circuit, and the source drain current vs time curve……………………………...…..12

Figure S13. The measured light power as a function of the polarization angle.............13

Figure S14. The responsivity of circular polarized light detector.................................14

Figure S15. The schematic and the photo of the single pixel imaging system..............15

Figure S16. Deviations of azimuth, ellipticity angle and degree of polarization….......16

Table S1. Performance comparison of intrinsic polarization photodetectors...............17

Reference……………………………………………………………………………..19

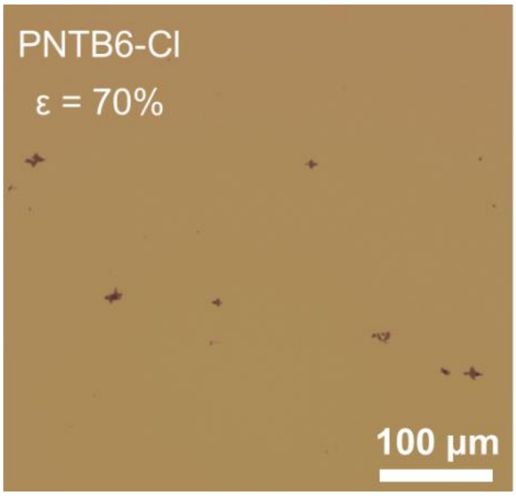

Figure S1. Microscope image of PNTB6-Cl film stretched under 70% strain.

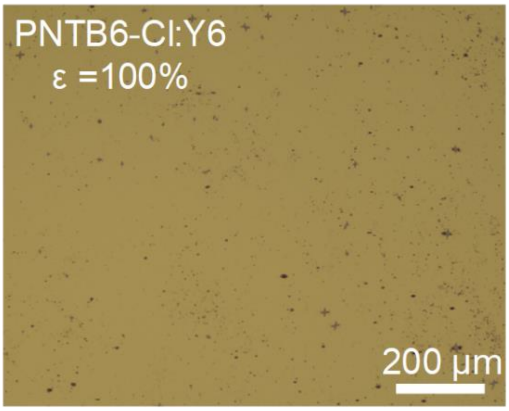

Figure S2. Microscope image of PNTB6-Cl film stretched under 100% strain.

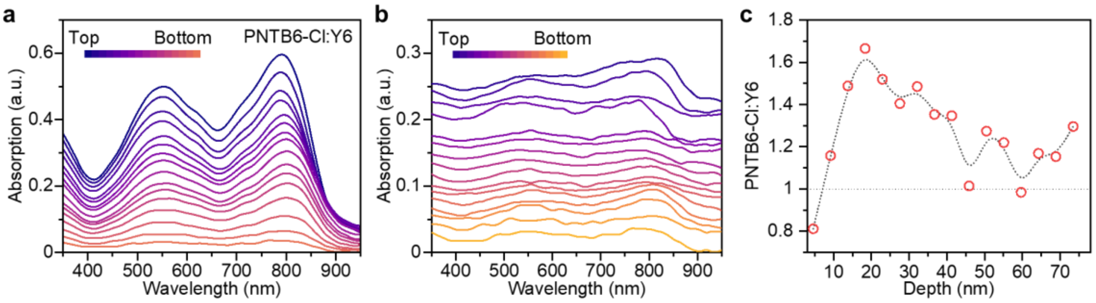

Figure S3. (a) Film-depth-dependent light absorption spectra of PNTB6-Cl:Y6 film. (b) The sublayer absorption calculated form film-depth-dependent light absorption spectra of PNTB6-Cl:Y6, the sublayer thickness corresponding to each spectrum is ~ 4-5 nm. (c) The calculated volume ratio of PNTB6-Cl and Y6 along the vertical direction from top to the bottom of PNTB6-Cl:Y6 film.

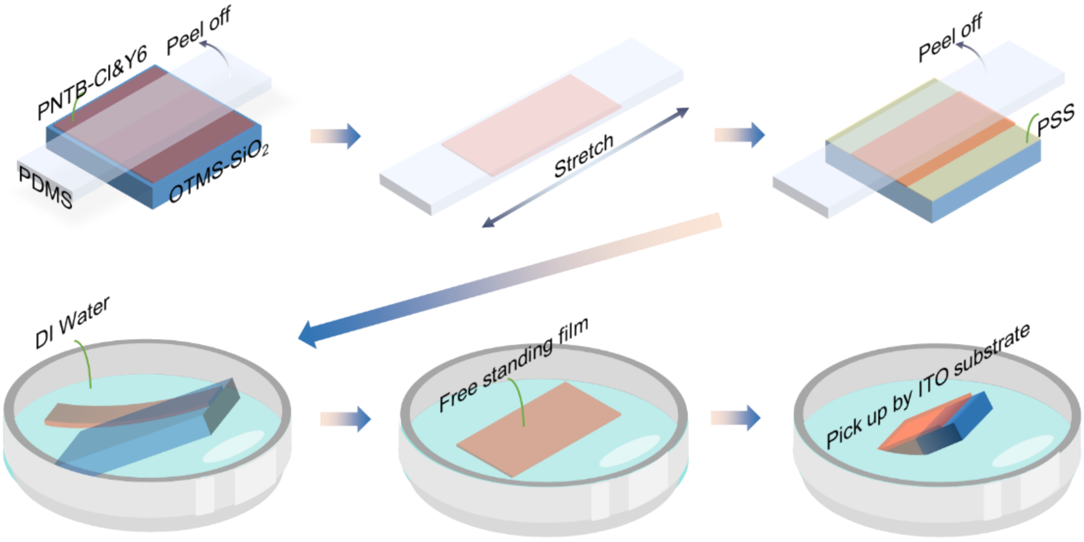

Figure S4. Manufacturing process of polarization organic photodetector.

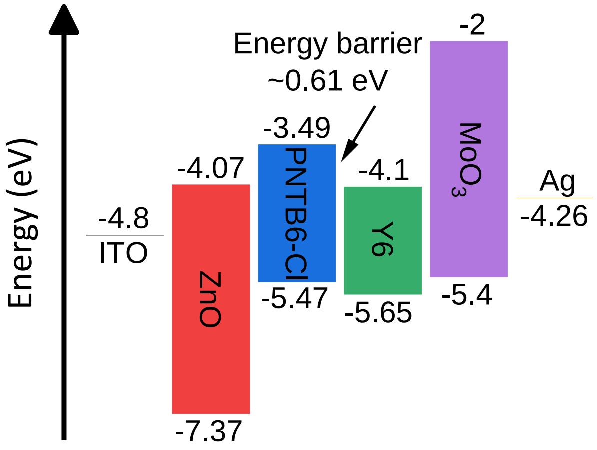

Figure S5. Schematic energy levels diagram of materials in photodetector.

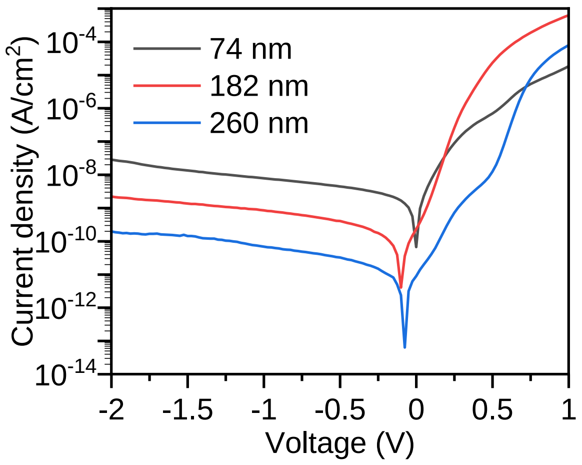

Figure S6. The dark current density of devices with the active layer thickness varying from 74 to 260 nm.

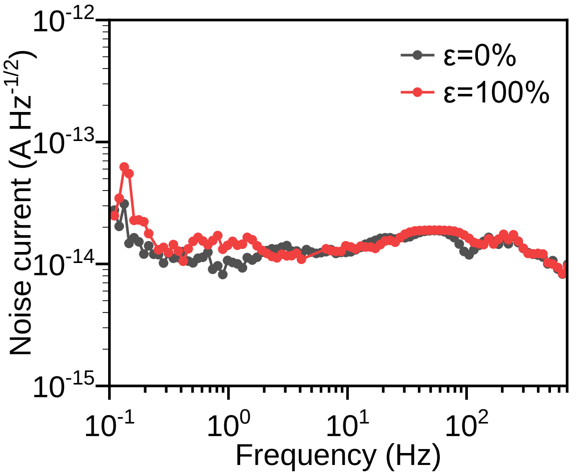

Figure S7. Noise spectra density of devices with unstrained and 100% strained film under 0 V.

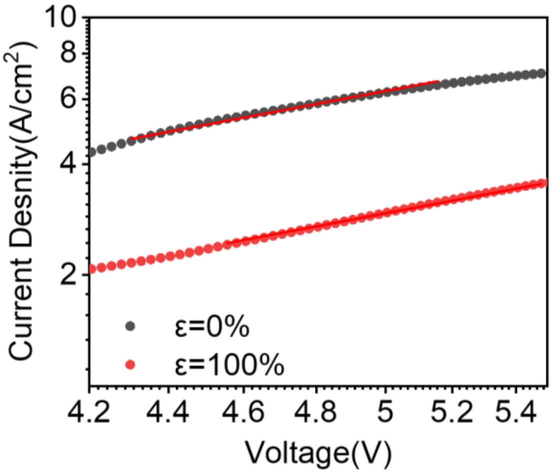

Figure S8. Current-voltage curves of hole-only devices with unstrained and 100% strained PNTB6-Cl:Y6 film.

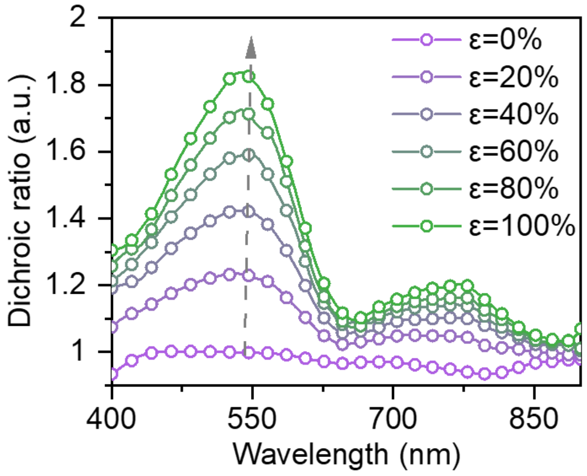

Figure S9. Dichroic ratio spectra of PNTB6-Cl:Y6 LBL film with strain varies from 0% to 100%, the step is 20%.

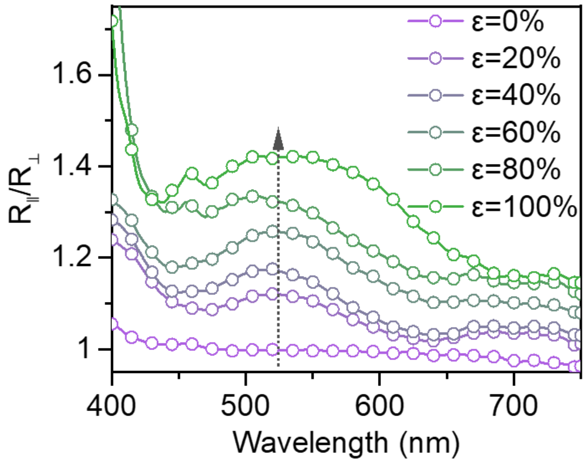

Figure S10. The responsivity anisotropy spectra of the film with strain varies from 0% to 100%, the step is 20%.

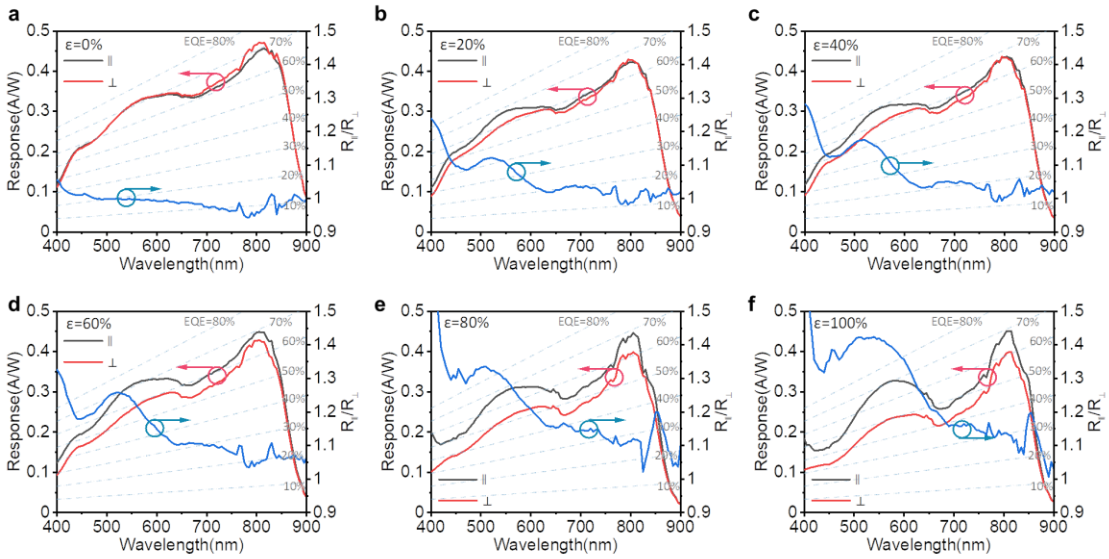

Figure S11. (a-f) The responsivities of the detectors under linear polarized light parallel (//) and perpendicular (⊥) to the strain direction with the film strained from 0% to 100%.

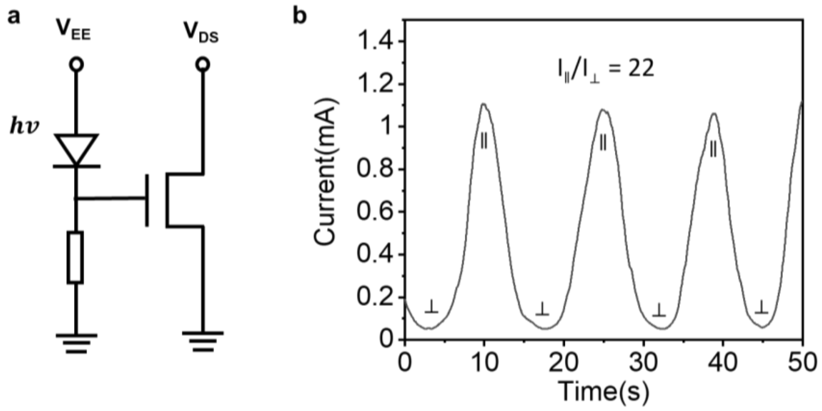

Figure S12. (a) Structure schematic diagram of the polarization-sensitive amplification circuit. (b) The source drain current vs time curve when rotate the polarization angle of the incident light.

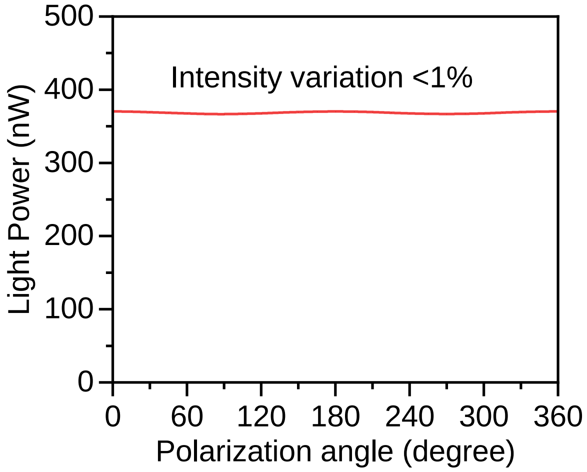

Figure S13. The measured light power as a function of the polarization angle under linear polarized light in polar curves test.

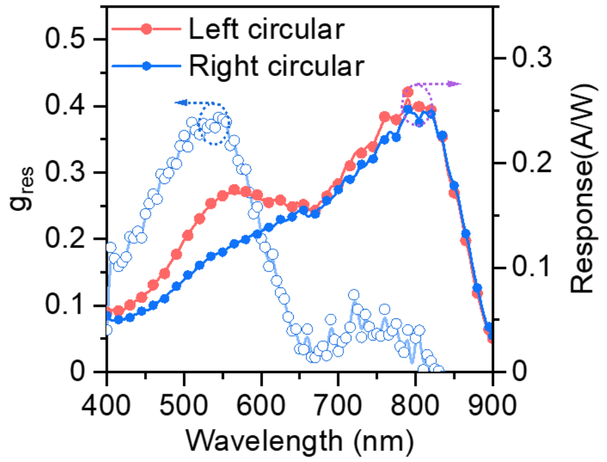

Figure S14. The responsivity of circular polarized light detector to left-handed circularly polarized light and right-handed circularly polarized light and the calculated g value as a function of wavelength.

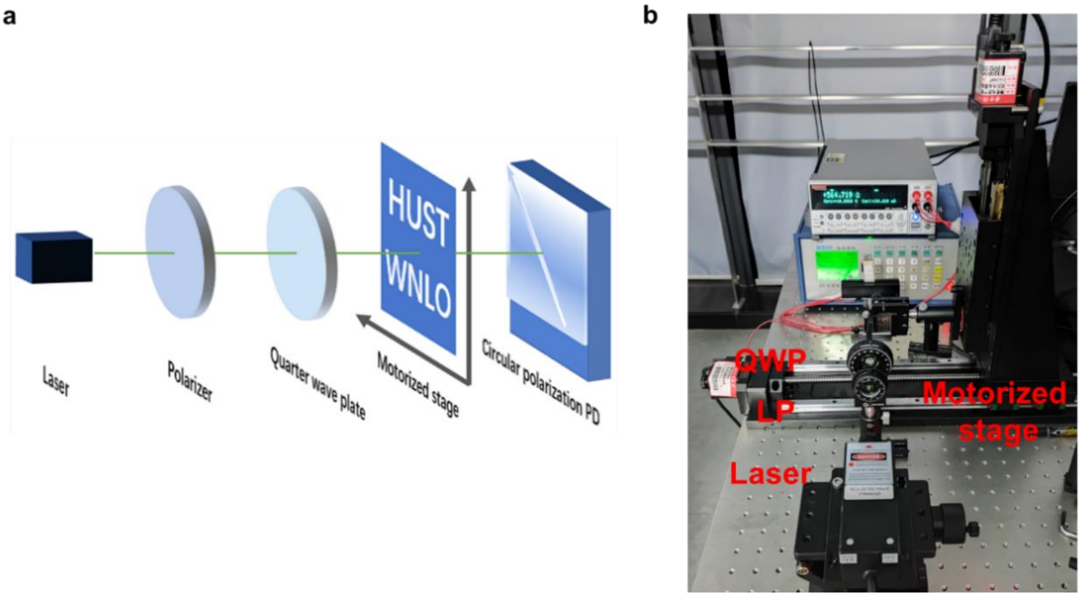

Figure S15. (a) The structure schematic and the (b) photo of the single pixel imaging system.

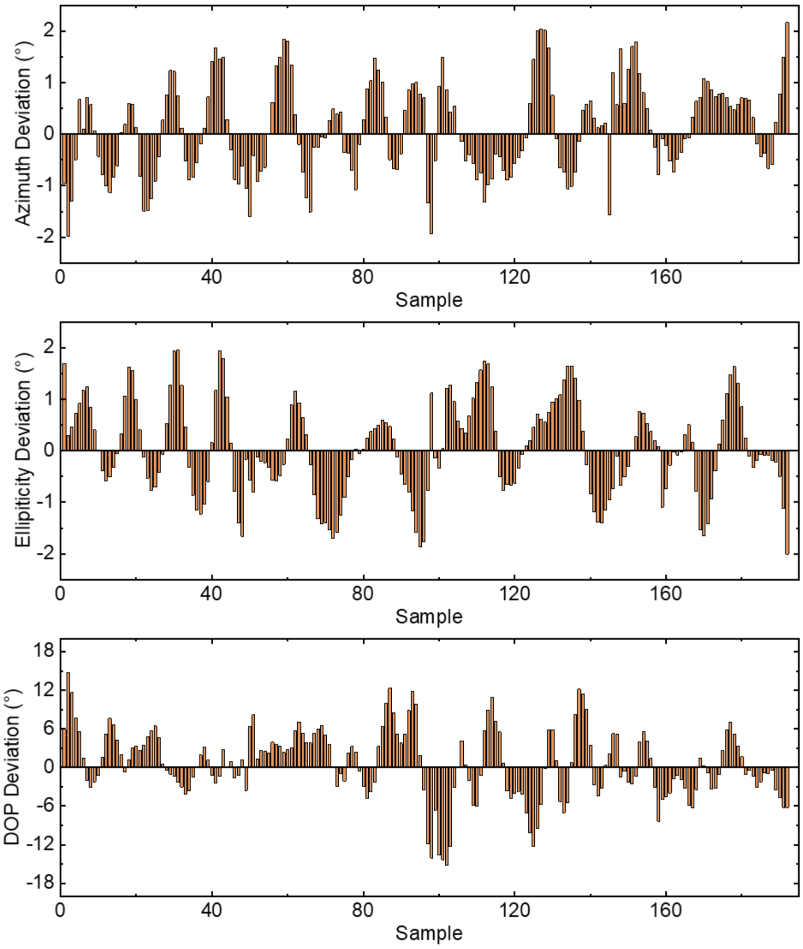

Figure S16. Deviations of azimuth, ellipticity angle and degree of polarization (DOP) between the retrieved and the generated SOPs.

Table S1. Performance comparison of intrinsic polarization photodetectors.

Material	Detectivity (Jones)	$R_{//}/R_{\perp}$	LDR (dB)	-3db (Hz)	Ref.
Black phosphorous /MoS_2	1.1×10^{10}	~30	~100	~9.5 k	[1]
WS_2/h-BN/$PdSe_2$	2.3×10^{10}	4.9	<20	~3.8 k	[2]
$MoTe_2/ReS_2$	1.00×10^{10}	1.03	~80	100 k	[3]
$WSe_2/ReSe_2$	1.00×10^{9}	2.09	~100	30 k	[4]
ZnSb	$1.70\times10^{10*}$	1.58	~26	~3	[5]
GaTe/MoS_2	$6.80\times10^{9*}$	2.9	~60	~34	[6]
TiS_3/Si	$1.04\times10^{10*}$	1.82			[7]
Sb_2Se_3	$4.40\times10^{9*}$	1.63		~5	[8]
ZrS3	$4.24\times10^{9*}$	2.55			[9]
$Sn^{II}Sn^{IV}S_3$	$2.70\times10^{10*}$	1.3		~2	[10]
SbI_3	$4.21\times10^{10*}$	1.16		~2	[11]
SbI_3/Sb_2O_3	$1.80\times10^{9*}$	3.14	~14	~2	[11]
Te	3.01×10^{9}	7.58	<20	~5 k	[12]
2D SiP_2	$6.00\times10^{11*}$	1.6			[13]
GeAs/InSe	2.00×10^{11}	18	~17	~1.4	[14]
Black Phosphorus	8.67×10^{8}	24		200 M	[15]
Black Phosphorus	1.27×10^{11}	288			[16]
$PdSe_2$	4.00×10^{7}	2.06		3.1 k	[17]
Bi_2Te_3/CuPc	1.85×10^{10}	2.03		~200	[18]
$(iBA)_2(MA)Pb_2I_7$	$1.00\times10^{11*}$	1.23		~1.1	[19]

$(iso\text{-}BA)_2PbI_4$	1.23×10^{10}	1.74	~70	~4	[20]
PBnDT-FTAZ:N2200	2.55×10^{10}	1.38	~60	500	[21]
PNTB6:Y6	6.1×10^{12}<br>1.24×10^{14}*	1.42	132	21.1 k	This work

*: The detectivity is calculated with the equation: $D_{sh}^{*} = \frac{R}{\sqrt{2qJ_d}}$, where q is elementary charge, which only consider the short noise, commonly the value will be around two orders of magnitude higher than the measured detectivity.

Reference

[1] J. Bullock, M. Amani, J. Cho, Y.-Z. Chen, G. H. Ahn, V. Adinolfi, V. R. Shrestha, Y. Gao, K. B. Crozier, Y.-L. Chueh, A. Javey, *Nat. Photonics* **2018**, *12*, 601.

[2] Y. Chen, Y. Wang, Z. Wang, Y. Gu, Y. Ye, X. Chai, J. Ye, Y. Chen, R. Xie, Y. Zhou, Z. Hu, Q. Li, L. Zhang, F. Wang, P. Wang, J. Miao, J. Wang, X. Chen, W. Lu, P. Zhou, W. Hu, *Nat. Electron.* **2021**, *4*, 357.

[3] J. Ahn, J.-H. Kyhm, H. K. Kang, N. Kwon, H.-K. Kim, S. Park, D. K. Hwang, *ACS Photonics* **2021**, *8*, 2650.

[4] J. Ahn, K. Ko, J. Kyhm, H.-S. Ra, H. Bae, S. Hong, D.-Y. Kim, J. Jang, T. W. Kim, S. Choi, J.-H. Kang, N. Kwon, S. Park, B.-K. Ju, T.-C. Poon, M.-C. Park, S. Im, D. K. Hwang, *ACS Nano* **2021**, *15*, 17917.

[5] R. Chai, Y. Chen, M. Zhong, H. Yang, F. Yan, M. Peng, Y. Sun, K. Wang, Z. Wei, W. Hu, Q. Liu, Z. Lou, G. Shen, *J. Mater. Chem. C* **2020**, *8*, 6388.

[6] J. Tan, H. Nan, Q. Fu, X. Zhang, X. Liu, Z. Ni, K. (Ken) Ostrikov, S. Xiao, X. Gu, *Adv. Electron. Mater.* **2022**, *8*, 2100673.

[7] Y. Niu, R. Frisenda, E. Flores, J. R. Ares, W. Jiao, D. Perez de Lara, C. Sánchez, R. Wang, I. J. Ferrer, A. Castellanos-Gomez, *Adv. Opt. Mater.* **2018**, *6*, 1800351.

[8] Z. Ma, S. Chai, Q. Feng, L. Li, X. Li, L. Huang, D. Liu, J. Sun, R. Jiang, T. Zhai, H. Xu, *Small* **2019**, *15*, 1805307.

[9] X. Wang, K. Wu, M. Blei, Y. Wang, L. Pan, K. Zhao, C. Shan, M. Lei, Y. Cui, B. Chen, D. Wright, W. Hu, S. Tongay, Z. Wei, *Adv. Electron. Mater.* **2019**, *5*, 1900419.

[10] H. Yang, L. Pan, X. Wang, H.-X. Deng, M. Zhong, Z. Zhou, Z. Lou, G. Shen, Z. Wei, *Adv. Funct. Mater.* **2019**, *29*, 1904416.

[11] M. Xiao, H. Yang, W. Shen, C. Hu, K. Zhao, Q. Gao, L. Pan, L. Liu, C. Wang, G. Shen, H.-X. Deng, H. Wen, Z. Wei, *Small* **2020**, *16*, 1907172.

[12] L. Tong, X. Huang, P. Wang, L. Ye, M. Peng, L. An, Q. Sun, Y. Zhang, G. Yang, Z. Li, F. Zhong, F. Wang, Y. Wang, M. Motlag, W. Wu, G. J. Cheng, W. Hu, *Nat. Commun.* **2020**, *11*, 2308.

[13] Z. Wang, P. Luo, B. Han, X. Zhang, S. Zhao, S. Wang, X. Chen, L. Wei, S. Yang, X. Zhou, S. Wang, X. Tao, T. Zhai, *ACS Nano* **2021**, *15*, 20442.

[14] J. Xiong, Y. Sun, L. Wu, W. Wang, W. Gao, N. Huo, J. Li, *Adv. Opt. Mater.* **2021**, *9*, 2101017.

[15] T.-Y. Chang, P.-L. Chen, J.-H. Yan, W.-Q. Li, Y.-Y. Zhang, D.-I. Luo, J.-X. Li, K.-P. Huang, C.-H. Liu, *ACS Appl. Mater. Interfaces* **2020**, *12*, 1201.

[16] S. Wu, Y. Chen, X. Wang, H. Jiao, Q. Zhao, X. Huang, X. Tai, Y. Zhou, H. Chen, X. Wang, S. Huang, H. Yan, T. Lin, H. Shen, W. Hu, X. Meng, J. Chu, J. Wang, *Nat. Commun.* **2022**, *13*, 3198.

[17] M. Dai, C. Wang, M. Ye, S. Zhu, S. Han, F. Sun, W. Chen, Y. Jin, Y. Chua, Q. J. Wang, *ACS Nano* **2022**, *16*, 295.

[18] M. Yang, J. Wang, Y. Zhao, L. He, C. Ji, H. Zhou, J. Gou, W. Li, Z. Wu, X. Wang, *ACS Nano* **2019**, *13*, 10810.

[19] Y. Liu, Z. Wu, X. Liu, S. Han, Y. Li, T. Yang, Y. Ma, M. Hong, J. Luo, Z. Sun, *Adv. Opt. Mater.* **2019**, *7*, 1901049.

[20] L. Li, L. Jin, Y. Zhou, J. Li, J. Ma, S. Wang, W. Li, D. Li, *Adv. Opt. Mater.* **2019**, *7*, 1900988.

[21] P. Sen, R. Yang, J. J. Rech, Y. Feng, C. H. Y. Ho, J. Huang, F. So, R. J. Kline, W. You, M. W. Kudenov, B. T. O'Connor, *Adv. Opt. Mater.* **2019**, *7*, 1801346.
